# Assessing the relationship between food insecurity and lifestyle behaviors among university students: a comparative study between Lebanon and Germany

**DOI:** 10.1186/s12889-023-15694-9

**Published:** 2023-05-03

**Authors:** Rana Rizk, Chadia Haddad, Hala Sacre, Diana Malaeb, Hanna Wachten, Jana Strahler, Pascale Salameh

**Affiliations:** 1 INSPECT-LB (Institut National de Santé Publique, d’Épidémiologie Clinique Et de Toxicologie-Liban), Beirut, Lebanon; 2 Department of Natural Sciences, School of Arts and Sciences, Lebanese American University, Byblos, Lebanon; 3 School of Medicine, Lebanese American University, Byblos, Lebanon; 4 Research Department, Psychiatric Hospital of the Cross, Jal Eddib, Lebanon; 5 School of Health Sciences, Modern University for Business and Science, Beirut, Lebanon; 6 School of Pharmacy, Lebanese International University, Beirut, Lebanon; 7 College of Pharmacy, Gulf Medical University, Ajman, United Arab Emirates; 8 Sport Psychology, Institute of Sport and Sport Science, Albert-Ludwigs-University Freiburg, Freiburg Im Breisgau, Germany; 9 Faculty of Pharmacy, Lebanese University, Hadath, Lebanon; 10 Department of Primary Care and Population Health, University of Nicosia Medical School, 2417 Nicosia, Cyprus

**Keywords:** Socioeconomic status, Food security, Lifestyle, Diet quality, Stress, Healthy eating pattern, Financial well-being, Mediterranean diet, Physical activity, Insomnia, Germany, Lebanon

## Abstract

**Background:**

Food insecurity is a common public health problem in both developed and developing countries. This study aimed to profile food insecurity among university students in a developed country with stable economic circumstances (Germany) and a developing Mediterranean country undergoing a severe economic and financial crisis (Lebanon) and examine the associations between food insecurity and lifestyle behaviors (i.e., physical activity, sleep, and adherence to a healthy eating pattern, such as the Mediterranean diet), stress, and financial well-being.

**Method:**

This online cross-sectional study was conducted between September 2021 and March 2022. Subjects were recruited through social media platforms (Facebook, WhatsApp, Instagram, and personal email) and in-class announcements by several university professors of various majors and from different universities in Lebanon and Germany. The final sample included 547 participants (197 from Lebanon and 350 from Germany).

**Results:**

Our findings showed a higher food insecurity rate in Lebanon compared with Germany (59% versus 33%). In the bivariate analysis, food insecurity was associated with insomnia (r = 0.230; *p* < 0.001) and stress (r = 0.225; *p* = 0.001); German university students had higher physical activity (*p* < 0.001), better diet quality (*p* < 0.001), and lower adherence to the Mediterranean diet (*p* < 0001) than Lebanese students. In the multivariable analyses, more stress was related to insomnia (B = 0.178; *p* < 0.001), while financial well-being was not associated with any of the lifestyle behaviors. Physical activity, insomnia, and Mediterranean diet adherence were not associated with the country or food insecurity (*p* > 0.05); however, living in Germany was associated with better diet quality (B = -7.85; *p* < 0.001).

**Conclusion:**

The high prevalence of food insecurity reported in this study is alarming, particularly among Lebanese students; German students had better diet quality and higher physical activity but worse adherence to the Mediterranean diet. Moreover, food insecurity was also associated with worse sleep and stress. Further studies are necessary to assess the role of food insecurity as a mediating factor between sociodemographic characteristics and lifestyle behaviors.

**Supplementary Information:**

The online version contains supplementary material available at 10.1186/s12889-023-15694-9.

## Introduction

Food security refers to having, at all times, physical, social, and economic access to sufficient, safe, and nutritious food that meets dietary needs and food preferences [1], whereas food insecurity results from limited or uncertain access to nutritionally adequate and safe foods [2–4]. While food insecurity is a common public health problem in developing countries [5], it is also on the rise in developed countries [6]. Experiencing food insecurity involves not having enough food, being unable to afford enough food, feeling anxious about affording meals, or having to resort to low-quality or unhealthy diets due to financial constraints [2]. The consequences of food insecurity include malnutrition, which can manifest as undernutrition, micronutrient deficiencies, or overweight and obesity [7].

University students are particularly vulnerable to food insecurity, with estimates ranging from 12.5% to 84% and an average rate of 42% [8]. With the rising costs of education and housing, university students facing budgetary constraints often prioritize spending on tuition, rent, and other utilities, leaving insufficient resources for food [9]. Compared with their food-secure counterparts, food-insecure students consume more processed “affordable” meals [10], fewer legumes, fruits, and vegetables (2.12 vs. 2.97 cup equivalents) [11], and have more occasional breakfasts (3.94 vs. 2.96 meals per week) and home-made meals (on 1.95 vs. 1.17 days per week) [12]; thus, they are less likely to adhere to healthy eating patterns [13]. Although there has been limited research on the associations between food insecurity and health and academic outcomes among university students, the few available studies suggest that food insecurity is linked to negative consequences in both domains. Specifically, food-insecure students have shown a decreased ability to concentrate [14] and lower grade point averages [14, 15] compared with their food-secure counterparts, with a 22% lower likelihood of having high GPAs of 3.5–4.0. Furthermore, food insecurity is associated with more than twice the odds of poor sleep quality [15], lower sleep quantity (3.64 vs. 2.87 of days of enough sleep) [16, 17] in the last week, and lower levels of physical activity (1.81 vs. 1.72 days of moderate- to vigorous-intensity physical activity in the last week) [16]. Generally, food-insecure students report increased stress, depression, and anxiety [15, 18, 19], which can further compound the detrimental effects of food insecurity on their lives. Most importantly, food insecurity can interfere with the health and lifestyle of university students, potentially leading to long-term consequences, such as increased risks of poor mental health and chronic diseases [16].

While previous studies have focused on characterizing food insecurity among university students within a single institution or limited settings, few studies have attempted to profile food insecurity and its correlates across different contexts. Such a comparison is crucial to elucidate whether experiences related to food insecurity among university students are universal or country-specific and subject to cultural artifacts. Furthermore, contrasting developed countries with developing ones rather than merely describing each country provides a deeper understanding of public health issues with a better investigation of regional differences. Hence, comparing food insecurity among university students in Germany and Lebanon was deemed essential. On the one hand, Germany, the most powerful economy in the European Union [20], is characterized by a stable political scene and one of the highest safety profiles in the world. On the other hand, Lebanon, a developing Mediterranean country, is currently facing one of the worst economic crises globally [21]. Several factors are heavily affecting the Lebanese population’s socioeconomic status and represent a principal source of stress, including the restrictions on bank withdrawals, which prevent people from accessing their savings, the currency devaluation of more than 90%, the soaring inflation [22], political turmoil, and security issues.

Given this context and to address the gaps in the literature, this study aimed to profile food insecurity among university students in Germany and Lebanon and examine the associations between food insecurity and factors that affect current and future physical and mental health, including lifestyle behaviors (i.e., physical activity, sleep, and adherence to a healthy eating pattern, such as the Mediterranean diet), stress, and financial well-being. The secondary objective was to assess the effect of the interaction between the country and food insecurity on lifestyle behaviors to explore whether food insecurity is associated with different restraints in health behaviors depending on the socioeconomic context. Findings from this study are expected to shed light on the food insecurity issue among university students and inform programmatic initiatives to promote a healthier university experience.

## Methods

### Design

This cross-sectional study used an online survey advertised in Lebanon and Germany and was announced as an exploration of “Food Insecurity, Eating Disorders, and Physical Activity Among University Student Populations in Lebanon and Germany”. Data collection took place between September 2021 and March 2022 amid a triple crisis in Lebanon, i.e., the COVID-19 pandemic, the unprecedented economic and financial crisis, and the repercussions of the Beirut port blast. The same period was characterized by a stable situation in Germany. Subjects were invited to participate in this study through advertisements on social media platforms (Facebook, WhatsApp, Instagram, and personal email) and in-class announcements by several university professors of various majors and from different universities in Lebanon and Germany. The online survey was created on Google Forms and the SoSci Survey platform [23] and required about 20 to 30 min to be completed. The questionnaire was available in English and German, assuming that university students in both countries were able to understand and answer the questions. Ten university students, five from each country, participated in a pilot phase to evaluate the clarity and acceptability of the questionnaire. Related data were incorporated into the final dataset, as they did not bias the outcome of the current investigation.

### Sampling and inclusion criteria

The study included participants currently enrolled university students (undergraduate or postgraduate), regardless of their major, and aged over 17 years. No further exclusion criteria were applied.

### Minimum sample size calculation

The minimum sample size was calculated using the G-Power software, version 3.0.10. The calculated effect size was 0.0526, expecting a squared multiple correlation of 0.05 (R2 deviation from 0) related to the Omnibus test of multiple regression [24]. The minimum necessary sample was *n* = 454, considering an alpha error of 5%, a power of 80%, and allowing 25 predictors to be included in the model [24]. An additional 20% of participants were targeted to account for potential missing values; the total achieved sample size was *n* = 547.

### Study variables

The survey included measures of food insecurity, financial well-being, dietary quality, and adherence to a healthy eating pattern [25], such as the Mediterranean diet, in addition to physical activity, sleep quality, stress, and sociodemographic characteristics.

#### Outcome variables: The Household Food Insecurity Access Scale (HFIAS)

Food insecurity in the past four weeks was assessed using the Household Food Insecurity Access Scale (HFIAS) [26]. This instrument assesses experiences of food insecurity (i.e., anxiety and uncertainty about the household food supply, insufficient quality, in addition to insufficient food intake and its physical consequences) with two sets of nine questions each, related to the occurrence and frequency-of-occurrence of food insecurity in the past four weeks, respectively. Occurrence questions are dichotomous (rated 0 = no and 1 = yes) [26], and each of these questions is followed by a frequency-of-occurrence question rated on a 3-point Likert scale (1 = rarely, 2 = sometimes, and 3 = often), documenting how often the specific condition occurred in the past four weeks [26]. The HFIAS score was calculated by summing the codes for each frequency-of-occurrence question, with a minimum possible score of 0 and a maximum possible score of 27, where higher scores indicate more food insecurity [26]. The score was finally dichotomized into food-secure and food-insecure categories following the algorithm provided by the tool developers [26]. This scale has been validated in Lebanon, where it demonstrated a Cronbach’s alpha of 0.91 and a moderate agreement in the test–retest reliability, with an ICC of 0.58 [27]. However, it has not yet been validated in Germany. In this study, the total sample had a Cronbach’s alpha of 0.920, with the German and Lebanese samples having Cronbach’s alpha values of 0.750 and 0.858, respectively.

#### Predictors’ variables

##### The Mediterranean Diet Adherence Screener (MEDAS)

Adherence to the Mediterranean diet (MD) was assessed using the Mediterranean Diet Adherence Screener (MEDAS), which could be considered a proxy for a healthy diet [28, 29]. The MEDAS was validated in Germany among 68 women and showed that agreement between the food frequency questionnaire (FFQ) and MEDAS was of a fair or better level (0.4 and larger values for agreement coefficients) for about half of the MEDAS questions; thus, the German MEDAS in its current version could be a helpful tool to assess adherence to MD [30]. In Lebanon, MEDAS has been used in several studies [31–33] but has not been validated. This 14-item questionnaire assesses the habitual frequency of consumption or the quantity consumed of 12 principal components and two food habits related to the Mediterranean diet. Answers in favor of the Mediterranean diet are scored one point, while unfavorable responses are graded zero, resulting in a score ranging from 0 to 14, with higher scores indicating higher adherence to the Mediterranean diet. In this study, Cronbach’s alpha was 0.390 in the total sample, 0.430 in the German sample, and 0.319 in the Lebanese sample.

##### The Rapid Eating Assessment for Participants-Shortened version (REAP-S)

The REAP-S is a 16-item validated tool used to assess diet quality [34, 35]. It has not yet been validated in Germany and Lebanon. The first 13 questions are rated on a 3-point Likert scale (usually/often = 1, sometimes = 2, and rarely/never or does not apply to me = 3). The total score ranges between 13 and 39, with a higher score indicating a higher diet quality. In this study, Cronbach’s alpha was 0.729 in the total sample, 0.649 in the German sample, and 0.740 in the Lebanese sample.

##### The Pittsburgh Sleep Quality Index (PSQI)

The Pittsburgh Sleep Quality Index (PSQI) was used to assess sleep quality during the past month. This validated tool comprises nine questions, with four examining sleep duration (usual sleep time, time to fall asleep, usual wake-up time, and actual sleep hours) and five exploring the reasons for sleep problems [36]. A previous study among 9284 community-dwelling adult German participants found good reliability (Cronbach’s alpha = 0.75) and validity of the PSQI, which was considered suitable for measuring sleep quality [37]. In Arab countries, the PSQI was validated among 35 healthy bilinguals, and it showed acceptable reliability (Cronbach’s alpha = 0.65) and a high convergent validity with the Insomnia Severity Index (r = 0.76) [38]. In this study, Cronbach’s alpha was 0.824 in the total sample, 0.791 in the German sample, and 0.836 in the Lebanese sample.

##### The International Physical Activity Questionnaire (IPAQ)-short form

Physical activity during the past seven days was assessed using the International Physical Activity Questionnaire (IPAQ)-Short Form [39]. In Lebanon, the IPAQ has been validated among 159 participants (students and staff members) from the Saint Joseph University of Beirut and showed high internal consistency (reliability), with Cronbach's alpha ranging from 0.769–1.00 (*p* < 0.001) and intraclass correlation coefficient (ICC) ranging from 0.625–0.999 (*p* < 0.001) [40]. The German version of the IPAQ has also been validated in adults, showing satisfactory properties [41]; a short version of the German IPAQ has also been validated in a small sample in Switzerland [42], while a modified long version has been validated in German adolescents [43]. This validated tool includes seven questions on the duration and frequency of physical activity categorized as light, moderate, and vigorous [44]. A logarithmic transformation was used to improve normality of the scale. The Metabolic Equivalent of Tasks (METs) were then calculated by multiplying the total minutes spent on the activity by the frequency and the constants of 3.3, 4.0, and 8.0 for light, moderate, and vigorous activities, respectively. The respective MET values for all activities performed in bouts of more than 10 min were then summed to result in total METs.

##### The InCharge Financial Distress/Financial Well-being (IFDFW) scale

Financial well-being was assessed using the InCharge Financial Distress/Financial Well-Being (IFDFW) Scale [45]. This tool is a validated 8-question self-reported measure with high internal consistency/reliability of perceived financial distress/well-being, ranging from overwhelming financial distress (lowest level of well-being) to no financial distress (highest level of well-being) [46]. This scale has been previously validated in Lebanon (Cronbach’s alpha was 0.925) [21] but not in Germany. In this study, Cronbach’s alpha was 0.850 in the total sample, 0.895 in the German sample, and 0.844 in the Lebanese sample.

##### The perceived stress scale

Stress over the past month was assessed using the 10-item Perceived Stress Scale (PSS-10), a 10-item validated tool that measures the perception of stress by describing respondents’ current life as unpredictable, uncontrollable, and stressful [47]. Answer options range from never (0) to very often (4), and the total score ranges from 0 to 40, where higher scores indicate higher perceived stress [48, 49]. This tool was validated in Germany among 2,527 participants and showed good internal consistency (Cronbach’s alpha = 0.84) and construct validity [50]. In Lebanon, it was validated among 268 women; the results showed that Cronbach’s alpha was 0.74 and a moderately high test–retest reliability, with Spearman’s correlation coefficient of 0.74 [51]. In this study, Cronbach’s alpha was 0.835 in the total sample, 0.860 in the German sample, and 0.756 in the Lebanese sample.

#### Covariate variables

The sociodemographic variables, such as age, gender, marital status, and employment status, were considered covariates.

### Statistical analysis

The SPSS software version 25 was used to analyze data. A descriptive analysis was carried out using numbers and percentages for categorical variables and means and standard deviations for continuous measurements. For the bivariate analysis, means were compared using the Student’s t-test after checking the normality of continuous variables and their variances homoscedasticity; percentages were compared using the Chi-square test when the expected cell count was higher than 5. In case the assumption was not met, the Wilcoxon and Fisher’s exact tests were used, respectively. Pearson correlation was used for assessing the linear correlation between continuous variables, and Fisher’s Z was used to compare correlation coefficients in a stratified analysis between countries. Coefficient values of │0.1–0.23│, │0.24–0.36│, and > │0.37│ indicated small, moderate, and large correlations, respectively [52]. A two-way ANOVA was used to compare the mean differences between two independent variables (country and food security). Four linear regression analyses were performed, taking the four lifestyle behaviors as the dependent variables. The independent variables were age, gender, marital status, employment, income level, PSS, IFDFW scale, country, and food security. The multi-collinearity was detected using the variance inflation factor (VIF) in the regression analysis models. Finally, a multilevel mixed-effect linear regression analysis taking the Gross Domestic Product (GDP) between countries as a random variable was conducted to evaluate the mean difference of the lifestyle variables on food security. A *p*-value less than 0.05 was considered significant.

## Results

### Sociodemographic characteristics of the sample population

A total of 547 participants were included in the analysis, with 197 (36.0%) from Lebanon and 350 (64.0%) from Germany. Table 1 provides detailed sociodemographic characteristics of the participants. Compared to the German sample, significantly higher proportions of participants in the Lebanese sample reported being food-insecure, single, unemployed, financially independent, receiving financial support, having no income, relying on parents or guardians for income, and living off campus with parents/guardians. Moreover, the mean BMI was significantly higher in the Lebanese sample. Conversely, a significantly higher proportion of students living in the city and having a higher average financial well-being was found in the German sample. More than 80% of the respondents in both samples were females.

**Table 1:** Comparison of sociodemographic characteristics in Lebanon and Germany

	**Lebanon (*n* = 197)**	**Germany (*n* = 350)**	**Pearson Chi-square**	***p*-value**
	**Frequency (%)**	**Frequency (%)**		
**Gender**				
Male	39 (19.8%)	63 (18.4%)	1.87	0.391
Female	158 (80.2%)	279 (81.6%)		
**Marital status**				
Single	168 (85.3%)	214 (62.0%)	59.52	** < 0.001**
Solid partnership/ not married	14 (7.1%)	122 (35.4%)		
Married	11 (5.6%)	9 (2.6%)		
Widowed	1 (0.5%)	0 (0%)		
Divorced	3 (1.5%)	0 (0%)		
**Financial Independence**				
Yes	62 (31.5%)	62 (18.6%)	11.53	**0.001**
No	135 (68.5%)	272 (81.4%)		
**Monthly income**				
No income	77 (39.1%)	86 (25.7%)	76.43	** < 0.001**
Low	48 (24.4%)	188 (56.3%)		
Intermediate	41 (20.8%)	56 (16.8%)		
High	31 (15.7%)	4 (1.2%)		
**Source of income**				
Parents or guardians	124 (62.9%)	134 (40.1%)	38.39	** < 0.001**
Parents plus own occupation	28 (14.2%)	129 (38.6%)		
Own occupation	45 (22.8%)	71 (21.3%)		
**Financial support**				
Yes	65 (33.0%)	64 (19.2%)	12.89	** < 0.001**
No	132 (67.0%)	270 (80.8%)		
**Employment status**				
Full-time employed	39 (19.8%)	15 (4.5%)	80.20	** < 0.001**
Part-time / marginally employed	35 (17.8%)	181 (54.2%)		
Not employed	123 (62.4%)	138 (41.3%)		
**Place of residence**				
On campus	6 (3.0%)	35 (10.5%)	183.00	** < 0.001**
Off campus, not with parents/guardians	15 (7.6%)	203 (60.8%)		
Off campus, with parents/guardians	176 (89.3%)	96 (28.7%)		
**Region of residence**				
In the city or rather urban	130 (66.0%)	281 (84.1%)	23.31	** < 0.001**
In the countryside or rather rural	67 (34.0%)	53(15.9%)		
**Household food insecurity**				
Food-secure	80 (40.6%)	232 (66.3%)	33.91	** < 0.001**
Food-insecure	117 (59.4%)	118 (33.7%)		
	**Mean ± SD**	**Mean ± SD**	**Effect size**	
**Age**	22.32 ± 5.14	22.87 ± 5.15	0.107	0.240
**BMI**	22.91 ± 4.29	22.09 ± 3.90	-0.203	**0.033**

### Descriptive statistics of the scales used in the study

Table 2 describes the medians, means, SDs, and ranges of the scales used in this study. The means of most scales were relatively low, as compared to the maximal values for every score, except for physical activity, healthy eating, and financial well-being, all of which had means above 50%.

**Table 2:** Descriptive summary of the used scales

	**Mean (SD)**	**Ratio of the scales (%)**^a^	**Median**	**Minimum score**	**Maximum score**
Household Food Insecurity Access Scale (HFIAS)	4.88 (4.44)	18.07%	3.00	1.00	21.00
International Physical Activity Questionnaires (IPAQ (log10))	3.25 (0.48)	77.01%	3.32	1.52	4.22
Pittsburgh Sleep Quality Index (PSQI)	6.79 (3.02)	32.33%	7.00	0	20.00
Mediterranean Diet Adherence Screener (MEDAS)	4.89 (2.05)	34.92%	5.00	0	11.00
Rapid Eating Assessment for Participants – Shortened Version (REAP-S)	30.82 (4.62)	79.02%	31.00	7.00	39.00
Perceived Stress Scale (PSS)	19.13 (6.63)	47.82%	19.00	2.00	40.00
InCharge Financial Distress/Financial Well-Being scale (IFDFW)	49.29 (15.07)	61.61%	47.00	8.00	80.00

^a^The percentage of mean was calculated by considering the mean values * 100 / the maximum theoretical value of the scales

### Bivariate correlations of the used scales

Table 3 shows the correlation between the used scales in the total sample and both countries. In the total sample, a positive correlation was found between food security and sleep quality (r = 0.230, *p* < 0.001) and stress (r = 0.225, *p* = 0.001). However, a negative correlation was found between financial well-being and food security (r = -0.219, *p* = 0.001).

**Table 3:** Pearson correlation analysis between the quantitative variables

**Total sample**							
	HFIAS	IPAQ	PSQI	MEDAS	REAP-S	PSS	IFDFW
HFIAS		0.007	0.230***	0.130	-0.136*	0.225**	-0.219**
IPAQ (Log10)			-0.073	0.083	0.095*	-0.160**	0.189***
PSQI				-0.075	-0.033	0.379***	-0.172**
MEDAS					-0.137**	0.016	-0.098*
REAP-S						-0.158**	0.291***
PSS							-0.396***
IFDFW							
**Stratified between countries**							
**GER**	HFIAS	IPAQ	PSQI	MEDAS	REAP-S	PSS	IFDFW
**LEB**							
HFIAS		-0.046	0.168	-0.034	-0.041	0.298**	-0.236*
IPAQ (Log10)	0.186*		< 0.001	0.172**	0.102	-0.020	< -0.001
PSQI	0.256**	-0.117		-0.094	-0.072	0.430***	-0.238***
MEDAS	0.165	0.166*	-0.051		0.022	-0.110	0.044
REAP-S	-0.016	-0.143	0.008	**-0.137**		-0.086	0.146*
PSS	0.063	-0.110	0.352***	0.021	0.047		-0.303***
IFDFW	0.052	0.099	-0.137	0.007	-0.010	-0.259***	

*IPAQ* International Physical Activity Questionnaires, *PSQI* Pittsburgh Sleep Quality Index, *MEDAS* Mediterranean Diet Adherence Screener, *REAP-S* Rapid Eating Assessment for Participants – Shortened Version, *PSS* Perceived Stress Scale, *HFIAS* Household Food Insecurity Access Scale, *IFDFW* InCharge Financial Distress/Financial Well-Being Scale

The upper half is the correlations from Germany and the lower half is the correlations from Lebanon.Values marked in Bold r > .02

^*^ < 0.05; ** < 0.01; *** < 0.001

When stratifying by countries, a positive association was found between stress and food security (r = 0.298, *p* = 0.002), and the association was negative between financial well-being and food security (r = -0.236, *p* = 0.015) within the German sample. A positive association was found between sleep quality and food security (r = 0.256, *p* = 0.005) in the Lebanese sample.

When comparing correlations between the two countries, the results showed that the association between food security and stress (r _Lebanon_ = 0.063, r _German_ = 0.298; Z = 2.724, *p* = 0.003) and food security and financial well-being (r _Lebanon_ = 0.052, r _German_ = -0.236; Z = 2.103, *p* = 0.018) were significantly marked in the German sample. The correlation between food security and sleep quality was shown in the Lebanese sample (r _Lebanon_ = 0.256, r _German_ = 0.168; Z = 1.029, *p* = 0.152).

### Bivariate analysis: correlates of lifestyle behaviors

Table 4 displays the bivariate analysis, taking lifestyle behaviors as the dependent variables.

**Table 4:** Bivariate analysis taking the lifestyle behaviors as the dependent variables

	**IPAQ (Log10)**	**PSQI total**	**MEDAS**	**REAP-S**
	**Mean** ± **SD**	**Mean** ± **SD**	**Mean** ± **SD**	**Mean** ± **SD**
**Gender**				
Male	3.39 ± 0.45	6.25 ± 2.87	4.98 ± 2.06	26.41 ± 6.40
Female	3.22 ± 0.48	6.91 ± 3.04	4.86 ± 2.05	26.15 ± 5.73
*p-value*	**0.006**	0.071	0.650	0.694
**Marital status**				
Single/ widowed/ divorced	3.23 ± 0.49	6.74 ± 2.86	4.94 ± 1.99	25.62 ± 6.03
Married/ Solid partnership	3.29 ± 0.44	6.93 ± 3.40	4.73 ± 2.21	27.65 ± 5.08
*p-value*	0.279	0.540	0.345	** < 0.001**
**Employment status**				
Not employed	3.15 ± 0.53	6.74 ± 3.09	4.97 ± 2.00	25.71 ± 5.99
Employed	3.35 ± 0.40	6.84 ± 2.94	4.81 ± 2.10	26.69 ± 5.67
*p-value*	** < 0.001**	0.721	0.400	0.058
**Monthly income**				
No income	3.22± 0.50	6.70 ± 2.92	4.83 ± 2.00	25.87 ± 6.03
Low	3.30 ± 0.44	6.79 ± 3.13	4.85 ± 2.19	27.22 ± 5.49
Intermediate	3.21 ± 0.52	6.74 ± 0.29	4.85 ± 1.88	24.83 ± 6.12
High	3.15 ± 0.47	7.41 ± 3.52	5.59 ± 1.70	24.88 ± 5.44
*p-value*	0.248	0.684	0.309	**0.002**
**Financial Independence**				
Yes	3.29 ± 0.44	6.86 ± 3.31	5.11 ± 1.90	25.99 ± 6.03
No	3.24 ± 0.49	6.77 ± 2.92	4.82 ± 2.09	26.27 ± 5.79
*p-value*	0.322	0.804	0.196	0.644
**Source of income**				
Parents or guardians	3.17 ± 0.53	6.77 ± 2.99	4.92 ± 1.98	25.62 ± 5.84
Parents plus own occupation	3.35 ± 0.39	6.68 ± 2.93	4.92 ± 2.20	27.73 ± 5.14
Own occupation	3.31 ± 0.43	6.98 ± 3.20	4.75 ± 2.03	25.46 ± 6.39
*p-value*	**0.003**	0.749	0.755	**0.001**
**Financial support**				
Yes	3.21 ± 0.52	7.19 ± 3.30	4.95 ± 1.98	25.35 ± 5.69
No	3.27 ± 0.46	6.66 ± 2.91	4.87 ± 2.07	26.48 ± 5.87
*p-value*	0.262	0.094	0.707	0.060
**Place of residence**				
On campus	3.08 ± 0.49	7.31 ± 4.05	4.30 ± 1.82	26.67 ± 6.03
Off campus, not with parents/guardians	3.37 ± 0.37	6.66 ± 2.80	4.70 ± 2.08	27.99 ± 4.88
Off campus, with parents/guardians	3.18 ± 0.53	6.84 ± 3.04	5.10 ± 2.03	24.74 ± 6.12
*p-value*	** < 0.001**	0.527	**0.034**	** < 0.001**
**Region of residence**				
In the city or rather urban	3.24 ± 0.47	6.71 ± 2.96	4.81 ± 2.01	26.36 ± 5.79
In the countryside or rather rural	3.28 ± 0.52	7.04 ± 3.19	5.13 ± 2.16	25.66 ± 6.01
*p-value*	0.451	0.312	0.161	0.256
	**Correlation coefficient**	**Correlation coefficient**	**Correlation coefficient**	**Correlation coefficient**
**Age**	0.038	0.052	0.057	-0.015
*p-value*	0.433	0.260	0.218	0.736
**IFDFW**	**0.189**	**-0.172**	**-0.098**	**0.291**
*p-value*	** < 0.001**	** < 0.001**	**0.037**	** < 0.001**
**BMI**	-0.023	0.089	0.055	**-0.117**
*p-value*	0.643	0.059	0.242	**0.012**

*IPAQ* International Physical Activity Questionnaires, *PSQI* Pittsburgh Sleep Quality Index, *MEDAS* Mediterranean Diet Adherence Screener, *REAP-S* Rapid Eating Assessment for Participants – Shortened Version, *IFDFW* InCharge Financial Distress/ Financial Well-Being Scale

r and p-values marked in bold are significant (*p* < 0.05)

A significantly higher mean IPAQ score was found among male, employed, and living off-campus participants. Also, those living with their parents (compared to other groups) had a significantly higher mean MEDAS score.

When taking the REAP-S as the dependent variable, a significantly higher mean REAP-S was found among married participants, those with low income, relying on their parents and own occupation for income, and living off-campus, not with parents/guardians.

Also, a positive correlation was found between financial well-being and physical activity and REAP-S scale, while a negative association was found between PSQI, MEDAS, and financial well-being. BMI was negatively correlated with the REAP-S scale.

### Combined effects of country and food security on lifestyle factors

**Fig. 1:**
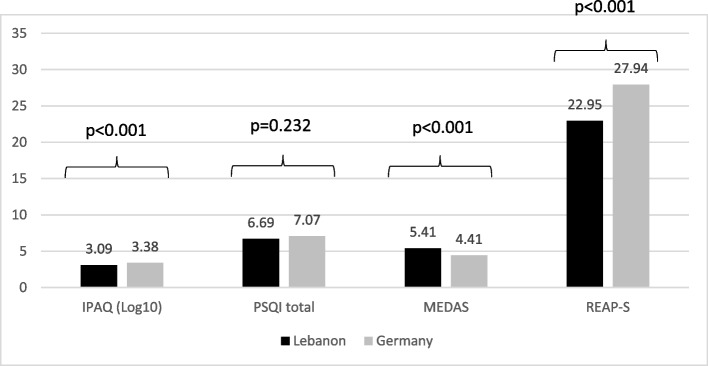
Mean of the total IPAQ, PSQI, MEDAS, REAP-S scales between the two countries; N_(Lebanon)_ = 197 and N_(Germany)_ = 350. (IPAQ International Physical Activity Questionnaires, PSQI Pittsburgh Sleep Quality Index, MEDAS Mediterranean Diet Adherence Screener, REAP-S Rapid Eating Assessment for Participants – Shortened Version)

**Fig. 2:**
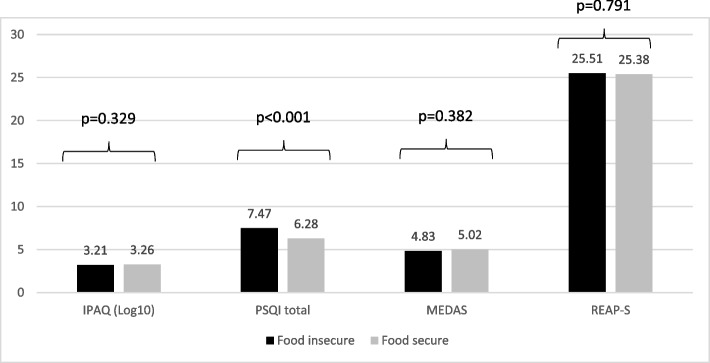
Mean of the total IPAQ, PSQI, MEDAS, REAP-S scales between the food security; N_(Lebanon)_ = 197 and N_(Germany)_ = 350. (IPAQ International Physical Activity Questionnaires, PSQI Pittsburgh Sleep Quality Index, MEDAS Mediterranean Diet Adherence Screener, REAP-S Rapid Eating Assessment for Participants – Shortened Version)

In the two-way ANOVA analysis conducted to examine the effect of country and food security on the overall lifestyle behavior scales, the main effect of the country appeared with all the lifestyle behavior scales except for the insomnia scale, where less physical activity, less healthy diet but more agreement with the Mediterranean diet was found in the Lebanese sample (Fig. 1). However, the main effect of food security was only found with the insomnia scale (Fig. 2), where food-insecure participants in both countries had higher insomnia (Table 5).

**Table 5:** Comparison of lifestyle behaviors between Lebanon and Germany

Variable	**Lebanon (*N* = 197)**	**Germany (*N* = 350)**	**Statistics of the two-way ANOVA analysis**
**IPAQ (Log10)**	3.09 ± 0.03	3.38 ± 0.03	F country (1, 430) = 29.87, ***p* < 0.001** F food secure (1, 430) = 0.95, *p* = 0.329
Food insecure	3.04 ± 0.53	3.38 ± 0.35	
Food secure	3.15 ± 0.56	3.37 ± 0.39	
**PSQI total**	6.69 ± 0.22	7.07 ± 0.23	F country (1, 466) = 1.43, *p* = 0.232 F food secure (1, 466) = 13.81, ***p* < 0.001**
Food insecure	7.40 ± 3.26	7.56 ± 3.24	
Food secure	5.99 ± 3.08	6.59 ± 2.73	
**MEDAS**	5.41 ± 0.14	4.41 ± 0.15	F country (1, 464) = 23.30, ***p* < 0.001** F food secure (1, 464) = 0.76, p = 0.382
Food insecure	5.43 ± 1.92	4.24 ± 1.73	
Food secure	5.45 ± 1.86	4.59 ± 2.14	
**REAP-S**	22.95 ± 0.38	27.94 ± 0.38	F country (1, 508) = 95.04, ***p* < 0.001** F food secure (1, 508) = 0.07, *p* = 0.791
Food insecure	23.57 ± 5.96	27.45 ± 5.52	
Food secure	22.33 ± 6.10	28.42 ± 4.48	

*IPAQ* International Physical Activity Questionnaires, *PSQI* Pittsburgh Sleep Quality Index, *MEDAS* Mediterranean Diet Adherence Screener, *REAP-S* Rapid Eating Assessment for Participants – Shortened Version

### Multivariable analysis

Four separate linear regression analyses were performed to investigate the associations between lifestyle behaviors and several demographic and socioeconomic factors. Each regression analysis had one of the four lifestyle behavior variables (IPAQ, PSQI, MEDAS, and REAP-S) as the dependent variable. The independent variables considered in the analyses were age, gender, marital status, employment status, income level, perceived stress, financial well-being, country, and food security.

In the first linear regression taking the IPAQ scale as the dependent variable the results showed that being employed (Beta = 0.21) was significantly associated with higher physical activity. However, being a female (Beta = -0.15) and having an intermediate income (Beta = -0.15) were significantly associated with lower physical activity. Considering the PSQI scale as the dependent variable, the results showed that higher stress (Beta = 0.17) was significantly associated with higher insomnia. Taking the MEDAS as the dependent variable, no significant association was found between the variables used and the adherence to the Mediterranean diet. Finally, no significant association was found between food security and the lifestyle behavior; also, the effect of the country was not significant with these variables except for the REAP-S scale, where being a German (Beta = -7.85) was associated with higher diet quality. Of note, when looking for the interaction between food security and country, no significant association was found for lifestyle behaviors (*p* > 0.05 for all), except for a trend related to diet quality (*p* = 0.054); nevertheless, stratifying the model by countries did not yield differential results (Table 6).

**Table 6:** Multivariable analysis

Linear regression analysis taking the lifestyle behaviors as the dependent variables					
	**Unstandardized Beta**	**Standardized Beta**	***p*-value**	**Confidence interval**	
				**Lower Bound**	**Upper Bound**
**Model 1 taking the IPAQ scale as the dependent variable**					
Age	-0.001	-0.009	0.863	-0.012	0.010
**Gender (Female vs male**^a^**)**	**-0.151**	**-0.121**	**0.009**	**-0.265**	**-0.038**
Marital status (Married vs single^a^)	-0.052	-0.048	0.329	-0.156	0.052
**Employment (employed vs unemployed**^a^**)**	**0.214**	**0.221**	** < 0.001**	**0.105**	**0.323**
Income (low vs no income^a^)	-0.094	-0.097	0.111	-0.211	0.022
**Income (Intermediate vs no income**^a^**)**	**-0.152**	**-0.121**	**0.049**	**-0.303**	**-0.001**
Income (high vs no income^a^)	-0.048	-0.024	0.649	-0.254	0.158
PSS scale	-0.002	-0.029	0.573	-0.010	0.005
IFDFW scale	0.001	0.025	0.676	-0.003	0.005
Country (Lebanon vs Germany^a^)	-0.042	-0.043	0.793	-0.356	0.272
Food insecure vs food secure^a^	0.165	0.164	0.332	-0.169	0.498
Country * Food security	-0.143	-0.364	0.166	-0.347	0.060
Adjusted R^2^ = 0.124					
**Model 2 taking the PSQI scale as the dependent variable**					
Age	0.042	0.066	0.196	-0.022	0.105
Gender (Female vs male^a^)	0.309	0.040	0.360	-0.354	0.972
Marital status (Married vs single^a^)	0.324	0.048	0.303	-0.294	0.943
Employment (employed vs unemployed^a^)	0.099	0.016	0.769	-0.562	0.759
Income (low vs no income^a^)	-.031	-0.005	0.930	-0.731	0.668
Income (Intermediate vs no income^a^)	-0.348	-0.044	0.449	-1.252	0.555
Income (high vs no income^a^)	0.329	0.028	0.599	-0.899	1.557
**PSS scale**	**0.178**	**0.389**	** < 0.001**	**0.134**	**0.221**
IFDFW scale	-0.012	-0.060	0.282	-0.034	0.010
Country (Lebanon vs Germany^a^)	-1.505	-0.245	0.110	-3.351	0.341
Food insecure vs food secure^a^	0.221	0.035	0.825	-1.745	2.188
Country * Food security	0.313	0.126	0.609	-0.888	1.513
Adjusted R2 = 0.164					
**Model 3: taking the MEDAS scale as the dependent variable**					
Age	0.041	0.093	0.087	-0.006	0.088
Gender (Female vs male^a^)	0.072	0.014	0.773	-0.420	0.565
Marital status (Married vs single^a^)	-0.034	-0.007	0.883	-0.491	0.422
Employment (employed vs unemployed^a^)	-0.223	-0.054	0.360	-0.701	0.255
Income (low vs no income^a^)	0.299	0.072	0.248	-0.209	0.807
Income (Intermediate vs no income^a^)	-0.050	-0.010	0.880	-0.706	0.605
Income (high vs no income^a^)	0.142	0.017	0.760	-0.767	1.050
PSS scale	-0.016	-0.050	0.334	-0.047	0.016
IFDFW scale	0.003	0.022	0.721	-0.013	0.019
Country (Lebanon vs Germany^a^)	0.646	0.155	0.352	-0.717	2.010
Food insecure vs food secure^a^	-0.792	-0.183	0.285	-2.246	0.662
Country ^a^ * Food security	0.393	0.231	0.385	-0.495	1.280
Adjusted R2 = 0.044					
**Model 4: Taking the REAP-S scale as the dependent variable**					
Age	-0.035	-0.028	0.570	-.155	0.085
Gender (Female vs male^a^)	-0.618	-0.041	0.331	-1.866	0.631
Marital status (Married vs single^a^)	0.770	0.059	0.194	-0.393	1.932
Employment (employed vs unemployed^a^)	0.038	0.003	0.951	-1.189	1.266
Income (low vs no income^a^)	-0.346	-0.029	0.605	-1.659	0.967
Income (Intermediate vs no income^a^)	-1.118	-0.074	0.193	-2.803	0.568
Income (high vs no income^a^)	1.188	0.052	0.306	-1.092	3.468
PSS scale	0.007	0.008	0.860	-0.074	0.089
IFDFW scale^a^	0.034	0.087	0.110	-0.008	0.075
**Country (Lebanon vs Germany**^a^**)**	**-7.850**	**-0.664**	** < 0.001**	**-11.311**	**-4.389**
Food insecure vs food secure^a^	-3.055	-0.250	0.104	-6.745	0.635
Country * Food security	**2.217**	**0.461**	**0.054**	**-0.034**	**4.469**
Adjusted R2 = 0.192					

The Independent variables entered in the four models: age, gender, marital status, employment, income level, PSS, IFDFW scale, country and food security

*IPAQ* International Physical Activity Questionnaires, *PSQI* Pittsburgh Sleep Quality Index, *MEDAS* Mediterranean Diet Adherence Screener, *REAP-S* Rapid Eating Assessment for Participants – Shortened Version, *PSS* Perceived stress scale,* IFDFW* InCharge Financial Distress/ Financial Well-Being Scale

^a^Reference group

^*^Interaction between country and food security

A multilevel mixed-effect linear regression analysis was conducted to check the possibility of a random effect of the country system on the dependent variables, taking the GDP as a random effect between the two countries. The results showed that the GDP was not associated with the lifestyle behaviors scales. Food security remained significant only with the PSQI scale, where food insecurity (Beta = 0.652) was associated with higher insomnia (Additional file 1: Appendix Table 1).

## Discussion

This study examined food insecurity among university students in Germany, a developed and stable country, and Lebanon, a developing country undergoing an unprecedented economic and financial crisis [53]. It explored the prevalence of food insecurity and evaluated its association with lifestyle behaviors, including physical activity, sleep, diet quality, and adherence to the Mediterranean diet, after adjustment over stress and financial well-being in both countries. Our findings showed a higher food insecurity rate in Lebanon compared with Germany (59% versus 33%).

Moreover, the bivariate analysis showed that food insecurity was associated with insomnia and stress, independent of the country profile. German university students had higher physical activity, better diet quality, and lower adherence to the Mediterranean diet than Lebanese students. In the current study, more stress was only related to insomnia, while financial well-being was not associated with any of the lifestyle behaviors. In the multivariable analyses, physical activity, insomnia, and Mediterranean diet adherence were not associated with the country or food insecurity; however, living in Germany was associated with better diet quality.

### Prevalence of food insecurity

Food insecurity is on the rise globally, with increasing rates being reported even in developed and economically stable countries [6, 54]. This public health problem has established itself at the center of health policy, given its devastating consequences on different population groups. Specifically, among university students, food insecurity might result from the rise in tuition fees at the university level, limited or insufficient financial aid, and high living costs, besides living alone without parents [55–57].

Food insecurity was observed in both countries, albeit it was more common in Lebanon compared to Germany. These results provide further evidence of this critical problem among university students in developing and developed countries [57–59]. It was anticipated that Lebanese students would exhibit a higher rate of food insecurity than their German counterparts (59.4% vs. 33.7% among their German counterparts), especially after experiencing the triple crisis since 2019 that involved the COVID pandemic, the deep economic and financial crisis, and the Beirut port explosion. More Lebanese university students were unemployed and without income compared with German students, which likely explains the higher prevalence of food insecurity in Lebanon. Factors driving food insecurity in Lebanon result in difficulty accessing appropriate food outlets, limited food knowledge, low income, and lack of social support [60], added to limited employment vacancies, poverty, and poor living conditions, all of which exacerbate the existing problem of food insecurity in the Middle East, where historically, rates are higher than in other regions [61].

### Lifestyle behaviors in Lebanon and Germany

In this study, physical activity level was higher in the German sample, in line with other local findings [60]; furthermore, younger German students were more physically active than the general population and other adults [62]. On the other hand, physical activity was reportedly lower in developing countries due to environmental factors, such as the absence or poor quality of sidewalks, parks/green spaces, and limited transportation facilities [63, 64]. Another reason underlying the higher engagement in physical activity among German students is the positive relationship between socioeconomic status and physical activity, where most German students in this study resided in the city and were wealthier than their Lebanese peers. A higher socioeconomic status enables individuals to participate in recreational or leisure-time physical activity and access facilities, resources, and better safety determinants [65, 66]. However, both samples had low sleep quality, a common finding among university students, who are at significant risk of having sleep issues as a response to high-stress periods [67].

As for diet, our findings suggest that diet quality was lower among Lebanese university students, most of whom were unemployed and had no or low income. Individuals classified in the low economic status may not prioritize diet quality as healthier foods are more expensive; hence, they are more likely to purchase cheap food, high in fat and sugar, instead of buying foods high in fiber, which in turn translates into a lower diet quality [68, 69]. Nevertheless, our results showed that adherence to the Mediterranean diet was lower among German participants, in line with the cultural differences in nutrition and eating behaviors, the German food being rich in energy and saturated fats and low in micronutrients [70]. These results, taken together, show that lower adherence to the Mediterranean diet does not necessarily mean a lower diet quality.

Indeed, compared with their Lebanese peers, German students had significantly higher diet quality, regardless of food insecurity levels. A recent study among 389 university students in Germany showed that the nutritional quality of their diet could be described as moderate according to the recommendations of the German Nutrition Society [71]. The study findings can be interpreted by the availability of canteens that offer the students a choice of a healthy dietary intake on campuses in Germany; thus, healthy foods might be more accessible to the population in Germany.

### Physical activity and food insecurity

The bivariate and stratified analyses showed that food insecurity was associated with physical activity in both countries, similar to previous findings where food insecurity was associated with less healthy physical activity among university students [16, 72, 73]. However, the association was not found in the multivariable analysis, possibly due to the small sample size or the lack of sensitivity of the physical activity questionnaire, while socioeconomic status and employment status were associated with physical activity; these factors would possibly play the role of mediators or confounders between food insecurity and physical activity. Additional research is necessary to depict this finding.

### Sleep and food insecurity

Food insecurity was found to be associated with insomnia in both bivariate and stratified analyses, consistent with previous findings [74, 75]. Food insecurity is likely to lead to insomnia, as food-insecure individuals, who do not have access to enough food, are more susceptible to experiencing fatigue, stress, anxiety, depression [17, 57, 76], and psychological distress in general [57], all of which disrupt the usual sleeping pattern and interrupt the physiological functioning [77]. Furthermore, food insecure individuals have limited knowledge, time, and resources to engage in healthy eating habits, resulting in increased consumption of saturated fats, added sugars, and refined grains, which is closely linked with obesity and thereby triggers poor sleep quality [78]. While a relationship with stress was visible, the association was not found in multivariable analysis; stress could thus be a mediator or a confounder between food insecurity and insomnia. This fact remains to be established by further studies.

### Diet and food insecurity

Our results showed that adherence to the Mediterranean diet was not correlated to food insecurity in both countries. A possible explanation could be the overall non-adherence to the Mediterranean diet among university students. This finding is not surprising in Germany but is unexpected in Lebanon. A study in Portugal found that food insecurity was associated with low adherence to the Mediterranean diet among 5653 participants [79], while in Greece, food insecurity was associated with lower adherence to the Mediterranean diet and the adoption of a cheaper and lower-quality diet among more food-insecure students [13]. Thus, Lebanese youth might be changing their overall diet and drifting away from the Mediterranean diet.

However, the multivariable analysis showed a trend for an interaction between the country and food insecurity, suggesting that food-insecure university students may sacrifice food quality to save money and choose less healthy options [80] differently by country; this result was also found in bivariate analysis among German students. Food-insecure individuals increase their consumption of calorie-dense, poor-quality foods and eat fewer fruits and vegetables [16, 81]. Our results are in line with the findings of two studies among adults and adolescents examining the associations between food insecurity and diet quality [82, 83]. They are also consistent with a systematic review, which included 16 studies and found lower intakes of healthy foods and higher intakes of unhealthy foods among food-insecure students [84]. The lack of this association among Lebanese students deserves to be further assessed.

### Implications

Our results highlight the importance of developing and implementing strategies on institutional levels to prevent and address food insecurity among university students. Food insecurity is a complex and multifaceted problem that should be tackled with tailored interventions at the individual, interpersonal, institutional, societal, and public policy levels, specifically through promoting sustainable food systems [85]. Integrating programs that provide access to food pantries, meal swipe plans, and food scholarships to university students is an effective strategy in this regard [86]. Applying systems to harmonize campus involvement with food assistance programs is also an effective method, as student involvement on campus will decrease behaviors associated with food insecurity [87, 88]. These recommendations are suggestive rather than conclusive, given that this issue has rarely been investigated in the international literature [89], warranting a call for evidence to support food security interventions in universities. Finally, university programs should also focus on promoting physical activity and healthy sleep behaviors.

### Strengths and limitations

This study contributes to the body of knowledge on food insecurity among university students. It utilized validated measures for food insecurity, diet, physical activity, and sleep and pioneered in investigating food insecurity in this population group through a comparative analysis between a developed and a developing country. Yet, this study has a limitation in that the majority of the tools used had not been validated in either both countries or one of them. While having validated tools would have enhanced the rigor of our methods, the acceptability of the data is not highly threatened with the stated tools, given that acceptable Cronbach alpha values reported in this study and the fact that all the tools used are internationally recognized, and the majority have been validated in countries with similar contexts to Lebanon and Germany [90–92]. Furthermore, the findings could not represent the general Lebanese and German populations as the study was conducted only on volunteer university students. Also, the samples from both countries were unequally distributed, with a majority of females, and the study did not consider the type and name of universities, which could have demonstrated differences in student economic classes and food security. A high collinearity was found between the country and food security in the regression analysis models, as calculated by the VIF, which might have led to lower precision coefficients. Another limitation is the risk of recall bias as a source of measurement error. Future studies among better-distributed samples (with higher percentages of males) should use more rigorous methods to assess diet as food records, physical activity as energy expenditure, and sleep through the sleep diary. Moreover, the type of university (public/private) was not recorded, although the snowball technique was applied to both public and private university students; however, this fact is expected to minimally affect our results, given that the socioeconomic status of the students was already measured through the financial wellness scale. Finally, there are shared concerns about challenges associated with online surveys, such as ineligible individuals posing as eligible subjects or multiple participation [93]. Although our experience in online surveys of university student populations in both countries does not reveal a high occurrence of these issues, one cannot overlook them.

## Conclusion

The high prevalence of food insecurity reported in this study is alarming, particularly among Lebanese students; German students had better diet quality and higher physical activity but worse adherence to the Mediterranean diet. Moreover, food insecurity was also associated with worse sleep and stress. Future research should assess the influence of the various mediating factors between student food insecurity and lifestyle behaviors, well-being, and success. These data would inform effective interventional programs and policies to alleviate food insecurity, optimize lifestyle behaviors, and improve the present and future of students.

## Supplementary Information

**Additional file 1: Appendix Table 1.** Multilevel mixed-effect linear regression analysis taking the lifestyle behaviors as dependent variables and the GDP as a random variable.

## Data Availability

The datasets generated during and/or analyzed during the current study are available from the corresponding author on reasonable request.

## Abbreviations

HFIAS: Household Food Insecurity Access Scale
MEDAS: Mediterranean Diet Adherence Screener
MD: Mediterranean diet
FFQ: Food frequency questionnaire
REAP-S: Rapid Eating Assessment for Participants-Shortened Version
PSQI: Pittsburgh Sleep Quality Index
IPAQ: International Physical Activity Questionnaire
ICC: Intraclass correlation coefficient
METs: Metabolic Equivalent of Tasks
IFDFW: InCharge Financial Distress/Financial Well-Being
PSS: Perceived Stress Scale
SPSS: Statistical Package for the Social Sciences
GDP: Gross Domestic Product
SD: Standard deviation
COVID: Coronavirus disease of 2019
