# Supplementary material for: Assessing the relationship between food insecurity and lifestyle behaviors among university students: a comparative study between Lebanon and Germany
Source: BMC Public Health. 2023 May 3;23:807. doi: 10.1186/s12889-023-15694-9 (PMC10154760; doi:10.1186/s12889-023-15694-9)
Supplement: Supplementary file 1 — Additional file 1: Appendix Table 1. Multilevel mixed-effect linear regression analysis taking the lifestyle behaviors as dependent variables and the GDP as a random variable. [file 12889_2023_15694_MOESM1_ESM.docx]

**Appendix**

| **Table 1: Multilevel mixed-effect linear regression analysis taking the GDP as a random variable** | | | | | |
| --- | --- | --- | --- | --- | --- |
| **Taking the IPAQ as the dependent variable** | | | | | |
|  | **Estimated Beta** | **P value** | **Confidence interval** | |  |
|  |  |  | **Lower** | **Upper** |  |
| Age | -.0007 | .884 | -.0114 | .009 |  |
| Gender (Female vs male*) | -.155 | **.006** | -.267 | -.043 |  |
| Marital status (Married vs single*) | -.0495 | .342 | -.152 | .052 |  |
| Income (low vs no income*) | -.085 | .143 | -.200 | .029 |  |
| Income (Intermediate vs no income*) | -.145 | .056 | -.294 | .003 |  |
| Income (high vs no income*) | -.054 | .596 | -.257 | .147 |  |
| Employment (employed vs unemployed*) | .208 | **<.001** | .100 | .315 |  |
| IFDFW | .0008 | .645 | -.002 | .004 |  |
| PSS | -.002 | .527 | -.009 | .004 |  |
| Food insecure vs food secure* | -0.065 | .218 | -.168 | .0385 |  |
| **Random effect of GDP** | **0.012** | **0.384** | **0.001** | **0.118** |  |
|  | | | | | |
| **Taking the PSQI as the dependent variable** | | | | | |
| Age | .0445 | .163 | -.018 | .107 |  |
| Gender (Female vs male*) | .327 | .326 | -.327 | .981 |  |
| Marital status (Married vs single*) | .390 | .205 | -.214 | .996 |  |
| Income (low vs no income*) | -.0175 | .960 | -.706 | .671 |  |
| Income (Intermediate vs no income*) | -.416 | .357 | -1.303 | .470 |  |
| Income (high vs no income*) | .181 | .767 | -1.018 | 1.381 |  |
| Employment (employed vs unemployed*) | .160 | .626 | -.485 | .806 |  |
| IFDFW | -.008 | .456 | -.0294 | .013 |  |
| PSS | .177 | **<.001** | .134 | .220 |  |
| Food insecure vs food secure* | .652 | **.037** | .040 | 1.264 |  |
| **Random effect of GDP** | **0.181** | **.466** | **.012** | **2.670** |  |
|  | | | | | |
| **Taking the MEDAS as the dependent variable** | | | | | |
| Age | .039 | .091 | -.006 | .086 |  |
| Gender (Female vs male*) | .077 | .754 | -.408 | .563 |  |
| Marital status (Married vs single*) | -.038 | .867 | -.486 | .409 |  |
| Income (low vs no income*) | .277 | .278 | -.224 | .778 |  |
| Income (Intermediate vs no income*) | -.058 | .858 | -.703 | .585 |  |
| Income (high vs no income*) | .185 | .684 | -.708 | 1.078 |  |
| Employment (employed vs unemployed*) | -.212 | .372 | -.680 | .255 |  |
| IFDFW | .002 | .806 | -.014 | .018 |  |
| PSS | -.014 | .347 | -.046 | .016 |  |
| Food insecure vs food secure* | **-.159** | .487 | -.608 | .290 |  |
| **Random effect of GDP** | **.306** | **.371** | **.034** | **2.745** |  |
|  | | | | | |
| **Taking the REAP-S as the dependent variable** | | | | | |
| Age | -.004 | .923 | -.102 | .092 |  |
| Gender (Female vs male*) | .242 | .639 | -.772 | 1.257 |  |
| Marital status (Married vs single*) | -.189 | .692 | -1.130 | .750 |  |
| Income (low vs no income*) | -.125 | .818 | -1.191 | .941 |  |
| Income (Intermediate vs no income*) | -.629 | .366 | -1.994 | .735 |  |
| Income (high vs no income*) | .132 | .888 | -1.711 | 1.976 |  |
| Employment (employed vs unemployed*) | .411 | .415 | -.578 | 1.400 |  |
| IFDFW | .001 | .925 | -.031 | .034 |  |
| PSS | -.115 | **.001** | -.182 | -.049 |  |
| Food insecure vs food secure* | -.656 | .172 | -1.599 | .286 |  |
| **Random effect of GDP** | .827 | .401 | .080 | 8.530 |  |
| Note: In the global model, the independent variable was food security, the random variable was the GDP and the covariates were age, gender, marital status, income, employment status, PSS, IFDFW.  GDP: Gross Domestic Product; IPAQ: International Physical Activity Questionnaires; PSQI: Pittsburgh Sleep Quality Index; MEDAS: Mediterranean Diet Adherence Screener; REAP-S: Rapid Eating Assessment for Participants – Shortened Version; PSS: Perceived Stress Scale; BDS: Beirut Distress Scale; HFIAS: Household Food Insecurity Access Scale; IFDFW: InCharge Financial Distress/Financial Well-Being Scale | | | | | |
| *Reference group | | | | | |
